# Topical Nifedipine Administration for Secondary Prevention in Frostbitten Patients

**DOI:** 10.3389/fphys.2020.00695

**Published:** 2020-06-19

**Authors:** Anna Carceller, Juan Pedro González Torcal, Ginés Viscor

**Affiliations:** Secció de Fisiologia, Departament de Biologia Cel⋅lular, Fisiologia i Immunologia, Facultat de Biologia, Universitat de Barcelona, Barcelona, Spain

**Keywords:** frostbite, topical nifedipine, secondary prevention, amputation, cold injuries

## Abstract

Frostbite is a cold-related injury with a growing incidence among healthy subjects. Sequelae after frostbite are frequent and vary among individuals. Here, we studied the thermal response in the digits of hands and feet of five subjects who had recovered from previous frostbite, except for their lasting sequelae. We considered three different conditions: digits unaffected by frostbite nor sequelae (healthy), those affected but which did not suffer amputation (frostbitten without amputation), and the remainder/stumps of digits that underwent partial amputation (frostbitten with amputation). Three consecutive immersions in cold water (8°C; 3 min) interspersed by 1 minute of thermal recovery were performed. After 30 min, a topical 10% nifedipine preparation was applied to hands and feet, and the same cold exposure protocol to evaluate its effect was followed. In basal condition and immediately after each immersion, the temperature of individual digits was assessed using thermography. We observed different thermal responses among the different digits of hands and feet, even without the nifedipine treatment. Nifedipine had a cooling effect on healthy and post-amputated tissue without thermal stress. In cold conditions, topic nifedipine application improved the cold response in healthy fingers but had a negative effect on those from which parts had been amputated. The topical nifedipine had detrimental effects on toes in all conditions. Topical nifedipine can help to the preservation of healthy fingers exposed to cold, with adequate thermal insulation; but it is necessary to remark its potentially harmful effects on previously frostbitten tissue. Because of the differences observed on individual regional response to cold, thermography can be a useful tool in the frostbite prevention for subjects habitually exposed to cold environment.

## Introduction

Frostbite is a local injury caused by exposure to environmental temperatures that are low enough to reach freezing point for a part of the body. The digits on hands (fingers) and feet (toes) are most affected by frostbite due to their peripheral location (Imray et al., 2009). Severity is related to the depth of tissue cooling, with a high incidence of digit total or partial amputation if the injury affects microvasculature and bone (Murphy et al., 2000). Sequelae after mild frostbite are frequent and can be debilitating, involving pain and hypersensitivity to cold that frequently persist for at least 4 years after the initial injury and can last for life (Ervasti et al., 2000; Hassi and Makinen, 2000; Carlsson et al., 2014). Severe frostbite, especially if acute treatment is delayed, can result in cell death and amputation, with functional sequelae.

Although a wide range of frostbite sequelae exists, for professional or personal reasons, some people previously affected by frostbite stay active in mountains or cold environments. These people face different degrees of symptoms in affected tissues on new exposure to low temperatures and a relative risk of a new frostbite incident. Nifedipine is an arterial specific vasodilator used in a variety of systemic pathologies in oral preparations, as well as topically for local symptoms in pathologies as Raynaud syndrome or chilblains. To our knowledge, no studies are available regarding its effect on tissue temperatures of frostbitten digits in the pathological spectrum (amputated and non-amputated). This study aims to elucidate whether a topical preparation based on nifedipine can relieve, or even reverse, cold-related symptoms in previously frostbitten extremities. This would have implications for those working in or exposing themselves to cold environments, and could minimize symptoms via a formulation that is easy to self-apply.

## Materials and Methods

### Subjects

Five subjects who had suffered moderate to severe frostbite (grades II, III, and IV) in recent years participated in the study, regardless of having undergone amputation of one or more digits or not. All of them suffered frostbite while practicing alpine activities, and after recovery, they continued regular activity in the cold for professional or sport reasons. Inclusion criteria were (1) healed from frostbite at least 1 year before data collection, (2) not have suffered any acute cold injury in the past year (including non-freezing injuries), (3) no local vasomotor alterations, such as Raynaud syndrome, and (4) no previous chronic illnesses. All of the recruited subjects described some degree of sequelae, from cold hypersensitivity to total intolerance to the exposure of the affected parts to extremely low environmental temperature. After approval by the local ethics committee, all of them were informed of the objective of the study and signed an informed consent form whereby they accepted to participate in the study. The protocol was conducted according to the principles of the Declaration of Helsinki.

### Thermography

**FIGURE 1:**
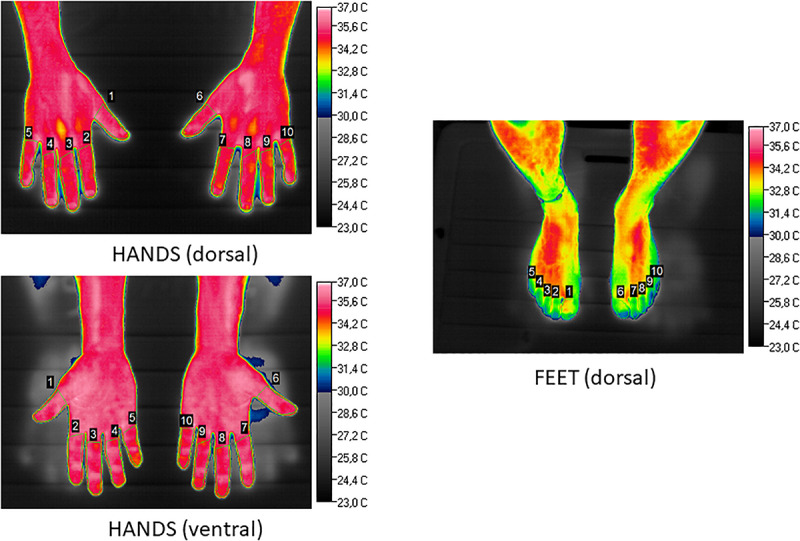
Illustration (basal conditions, Subject 2) of selected areas for calculation of minimal, maximal and average temperature of each finger (dorsal and ventral surfaces) and the dorsal surface of feet. Temperature scale (°C) is to the **right** of each picture.

An infrared thermography camera was used to assess surface skin temperature (NEC^®^ H2640, Avio Infrared Technologies Co., Tokyo, Japan), with a thermal sensitivity of 0.03°C. All measurements were performed using low-infrared-emission foam as a background. The test was performed in a room at a constant temperature of 23°C, with no direct sunlight so as not to alter the precision of the measurements. Infrared data from the images were processed using the thermographic image analyzer Image Processer Pro II. A trained observer checked the delimitation of the perimeter of the fingers, and the software automatically computed the average, maximal and minimal temperature for the selected areas of each digit in all the images. Figure 1 contains representative images showing the delimited areas for the calculation of average, maximal and minimal temperatures of each digit.

### Protocol

After 30 min of thermal acclimatization to room temperature, basal temperature image was taken, and immediately followed by 3 immersions of 3 min each in a tank filled with cold water at a constant temperature of 8°C (Keramidas et al., 2015). The time elapsed between these consecutive immersions was 1 minute. After the third immersion, a Vaseline-based cream containing 10% nifedipine was applied to the hands and feet of each participant. After a further 30 min at ambient temperature rewarming, 3 new immersions of 3 min each, again interspersed by 1 min of incomplete recovery, were performed in the cold water (8°C). Thermographic images of the extremities were taken immediately after each immersion. The subjects were asked not to move their limbs, while their hands and feet were dried without rubbing.

### Statistics

Because maximal and minimal temperatures offered considerable statistical dispersion, average local temperatures for each of the twenty digits (hands and feet) were assessed separately for statistical calculations, with the exception of one of the subjects who only participated with upper limbs, because of recent frostbitten injuries sustained to the feet. Thus, we studied a total number of 90 digits. We considered healthy (HD, *n* = 47), frostbitten non-amputated (FNA, *n* = 35) and frostbitten amputated (FA, *n* = 8) digits separately, in order to elucidate possible differences in their patterns of response. The total of healthy (*n* = 47) and affected (*n* = 43) digits are balanced. In order to minimize possible inter-subject and intra-subject vasomotor response variability, each subject was considered as his or her own control in basal condition. Statistical analysis was performed by comparing the difference in the decay from basal temperature along consecutive immersions for each digit in each participant, instead of the absolute temperature measured, to avoid the effect of different basal thermal conditions between subjects. A three way RM-ANOVA was applied. By comparing the effect of the treatment in the same subject and under the same conditions, changes in the thermal response can be attributed only to nifedipine application thus minimizing other possible confounding factors. *Post hoc* analysis (Student’s *t*-test for repeated measures) was performed considering step changes for individual digits into the three above mentioned categories (HD, FNA, FA), thus allowing to contrast the thermal responses of each clinical condition. Statistical significance was set at *p* < 0.05 for all the analysis, which we performed using SPSS v.15 (SPSS Inc., Chicago, United States).

## Results

Temperatures were computed from thermographies for each finger and toe to check the local thermal response (Figure 1), from basal conditions, during the cooling protocol, with and without nifedipine treatment. This yielded a total of *n* = 1,130 processed values.

The average basal temperature before the cooling protocol was 30.7°C ± 0.2°C (CI: 95%). Average temperatures for all measurements after first, second and third immersion, regardless of the presence or not of the nifedipine treatment, were 17°C ± 0.17°C, 14.8°C ± 0.17°C, and 13.8°C ± 0.17°C, respectively (CI: 95%).

**FIGURE 2:**
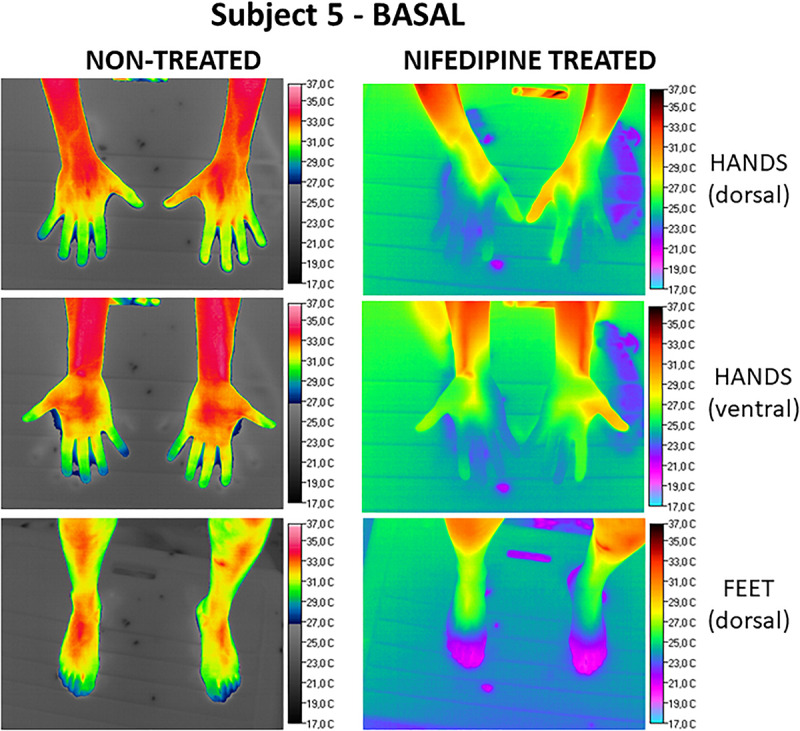
Comparative effect under basal conditions (Subject 5) of nifedipine on the surface temperature of hands (dorsal and ventral view) and feet. Images obtained without nifedipine treatment are on the **left**. The **right** panel shows the images registered after nifedipine treatment. Temperature scale (°C) is to the **right** of each picture.

**FIGURE 3:**
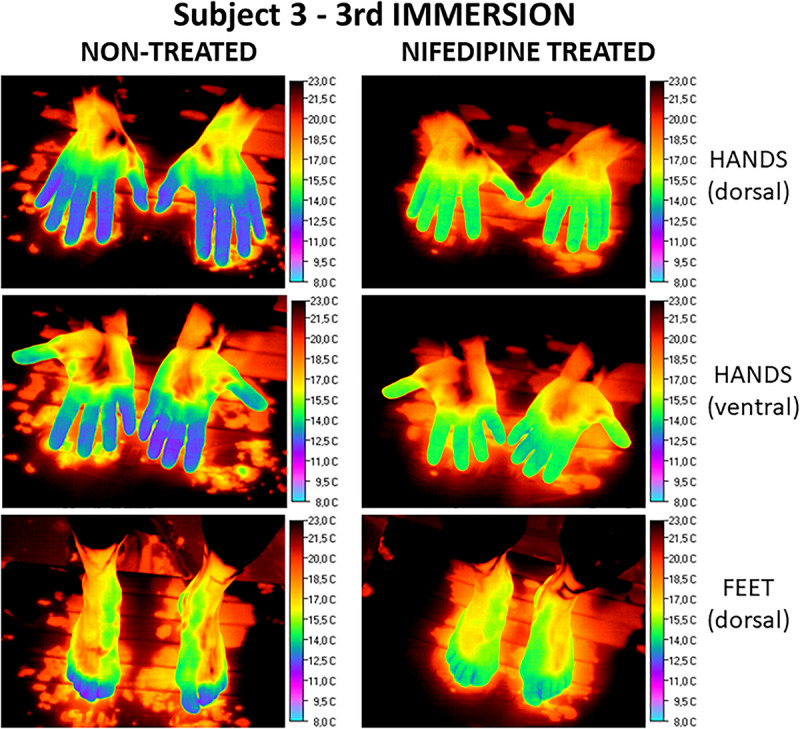
Comparative effect after the third immersion (Subject 3) of nifedipine on the superficial temperature of hands (dorsal and ventral view) and feet. Images obtained without nifedipine treatment are on the **left**. The **right** panel shows the images registered after nifedipine treatment. Temperature scale (°C) is on the **right** of each picture.

Regarding the global response of fingers and toes, we found, as expected, due to their lower surface to volume ratio, that thumbs were the digits that maintained the highest average temperatures (21.3°C ± 0.3°C right and 21.5°C ± 0.3°C left hand, respectively), with statistically significant differences when compared to all the other fingers and toes. The coldest digit was the middle finger, with global values of 19.8°C ± 0.3°C for both hands, although differences when comparing with the rest of the digits did not reach statistical significance. Toes showed globally cooler temperatures than hands. Figure 2 illustrates the effect of the nifedipine treatment under basal conditions on Subject 5. Meanwhile, Figure 3 presents the comparative effect of nifedipine treatment on the temperature of the fingers and toes of Subject 3 after the third immersion.

### Comparison Between Hands and Feet

The anatomical thermal pattern of the hands was not the same as in the feet: the big toe showed greater temperatures than the others, averaging 18.6°C ± 0.4°C on the right foot and 18.7°C ± 0.4°C on the left. The coolest toe was the second one (2 and 7 in Figure 1), with average temperatures of 17.3°C ± 0.4°C for the right foot and 17.1°C ± 0.4°C for the left.

Considering only the hand measurements, average finger basal temperature was 32.1°C ± 0.2°C and average temperature after first, second and third immersions was: 17.5°C ± 0.2°C, 15.5°C ± 0.2°C, and 14.4°C ± 0.2°C, respectively. Computing all the data for all the conditions, healthy digits (HD) showed an average temperature of 18.9°C ± 0.1°C, frostbitten non-amputated fingers (FNA), 20.3°C ± 0.1°C and frostbitten amputated (FA), 20.4°C ± 0.6°C. The difference of temperature between healthy and frostbitten digits reached statistical significance (*p* < 0.05), regardless of having undergone amputation or not.

### Comparison of Digit Conditions

When classifying according to the condition of the digits, average temperature of HD was of 17.9°C ± 0.1°C. Regarding frostbitten parts, FA digits presented an average temperature of 20.4°C ± 0.3°C and FNA 18.9°C ± 0.1°C (CI 95%).

When comparing the response to cold of healthy digits versus frostbitten digits, there were no significant differences in basal temperatures, although significant differences were reached when comparing frostbitten digits with healthy ones after maximal cooling (third immersion), with frostbitten ones being warmer than healthy digits (Table 1). When considering the interaction between response to cold and nifedipine treatment for HD, FA and FNA, statistically significant differences were only found in amputated toes, where treatment with nifedipine showed lower temperatures than when no treatment was applied (Table 1).

**TABLE 1:** Average temperatures (°C) of healthy or frostbitten fingers and toes under basal and maximal cooling (after third immersion) conditions, without nifedipine treatment.

		**Healthy (*n* = 47)**	**Frostbitten (*n* = 43)**	***p***
Fingers	Basal	33.1 ± 1.9	33.2 ± 0.9	NS
	Maximal cooling	13.9 ± 1.2	14.9 ± 0.2	0.04
Toes	Basal	33.1 ± 2.2	34.2 ± 0.5	0.04
	Maximal cooling	12.3 ± 3.8	14.1 ± 0.9	0.04

### Comparison Between Condition and Treatment

When the effects of treatment were assessed by pooling measurements for all conditions, average temperature without nifedipine was of 19.9°C ± 0.1°C and with nifedipine, 18.3°C ± 0.14°C (CI: 95%).

Significant differences were found when comparing previously frostbitten fingers without amputation under basal conditions, which were warmer after nifedipine administration. In contrast, after maximal cooling, healthy digits were significantly less cold after application of the treatment; whereas amputated digits lost more temperature when treated with the nifedipine cream (Tables 2, 4).

**TABLE 2:** Effects of the treatment with nifedipine on the temperature (°C) of fingers (average for both hands) considering digit condition.

	**Basal**			**Maximal cooling**		
	**HD (*n* = 47)**	**FNA (*n* = 35)**	**FA (*n* = 8)**	**HD (*n* = 47)**	**FNA (*n* = 35)**	**FA (*n* = 8)**
No Treatment	33.1 ± 1.9	33.6 ± 1.4	33.1 ± 0.9	13.9 ± 1.2	14.9 ± 2.0	14.9 ± 0.4
Nifedipine	27.4 ± 3.5	34.1 ± 0.8	33.1 ± 0.3	14.3 ± 0.7	13.1 ± 0.6	12.8 ± 0.8
*p*-value	ns	**0.007**	ns	**0.0009**	ns	**0.036**

*All measurements are mean ± SD. HD, healthy digits; FNA, frostbitten non-amputated; FA, frostbitten with amputation. Bolded values are statistically significant (*p* < 0.05).*

Regarding toes, significant differences were only reached in healthy digits when the basal measurement was performed, with those toes that did not receive treatment being warmer. Amputated toes, after nifedipine topical cream application, were significantly cooler both in basal measurements and after maximal cooling (Tables 3, 4).

**TABLE 3:** Effects of nifedipine treatment on the temperature (°C) of toes (average for both feet) considering digit condition.

	**Basal**			**Maximal cooling**		
	**HD (*n* = 47)**	**FNA (*n* = 35)**	**FA (*n* = 8)**	**HD (*n* = 47)**	**FNA (*n* = 35)**	**FA (*n* = 8)**
No treatment	33.1 ± 2.2	31.1 ± 2.7	34.1 ± 0.5	12.3 ± 1.1	12.6 ± 1.6	14.1 ± 0.8
Nifedipine	21.3 ± 0.4	23.7 ± 4.7	25.7 ± 4.4	13.2 ± 1.3	12.7 ± 0.8	12.3 ± 0.8
*p*-value	**0.00001**	ns	**0.0009**	ns	ns	**0.008**

*All measurements are mean ± SD. HD, healthy digits; FNA, frostbitten non-amputated; FA, frostbitten with amputation. Bolded values are statistically significant (*p* < 0.05).*

**TABLE 4:** Effects of nifedipine treatment on the drop in temperature (°C) of fingers and toes from the basal condition (without nifedipine treatment) after the first (1st), second (2nd), and third (3rd) immersions.

			**No treatment**			**Nifedipine**		
	**Position**	**Immersion**	**HD**	**FNA**	**FA**	**HD**	**FNA**	**FA**
			**(*n* = 47)**	**(*n* = 35)**	**(*n* = 8)**	**(*n* = 47)**	**(*n* = 35)**	**(*n* = 8)**
Hands	Dorsal	Basal	–	–	–	−7.21.7	−1.11.7	0.40
	Ventral		–	–	–	−6.41.0	−0.33.2	−0.50
Feet	Superior		–	–	–	−11.02.6	−7.84.5	−10.64.1
Hands	Dorsal	1st	−16.32.0	−17.52.4	−12.80	−8.90.0	−15.92.1	−14.20
	Ventral		−15.81.5	−15.41.9	−15.40	−8.90.3	−15.70.9	−15.60
Feet	Superior		−15.92.4	−15.41.4	−15.42.3	−6.11.8	−8.43.0	−7.02.8
Hands	Dorsal	2nd	−18.41.6	−19.42.0	−16.80	−10.40.3	−18.01.8	−18.80.0
	Ventral		−17.31.0	−18.52.3	−18.60	−10.40.1	−19.02.4	−19.70.0
Feet	Superior		−19.02.8	−18.92.9	−19.21.0	−7.82.0	−11.24.8	−9.34.0
Hands	Dorsal	3rd	−11.713.0	−14.29.4	−17.30.0	−11.00.5	−19.12.4	−19.60.0
	Ventral		−10.912.7	−13.83.9	−19.20.0	−11.10.1	−18.22.6	−21.10.0
Feet	Superior		−15.310.2	−14.611.1	−20.81.5	−8.01.7	−12.26.2	−10.75.1

*HD, healthy digits; FNA, frostbitten non-amputated; FA, frostbite with amputation; NFD, treated with nifedipine; NOT, no treatment.*

## Discussion

Frostbite is a cold-related pathology that involves local cell damage and ischemia, being especially frequent in digits, due to its distal location. We found different thermal responses during fast cooling between hands and feet: hands being warmer, especially the thumb. In toes, the biggest one was the coldest.

Applying a topical preparation of nifedipine leaded to lower digit temperatures in healthy tissues when not exposed to cold, both in hands and toes. Use of topical nifedipine can therefore be considered as unsafe in the absence of cold stress, as it impairs the normal thermoregulatory systems of healthy limbs. This can be extrapolated to amputated toes that conserve the neurovascular system intact. Conversely, regarding the cold response of healthy fingers, we found significantly warmer temperatures when using nifedipine. Regarding toes exposed to the cold, a detrimental effect was found in amputated limbs.

Exposure of a person to cold temperatures implies peripheral vasoconstriction in order to reduce heat loss and maintain core temperature. Vasoconstriction can be as severe as to induce cell death in those distal parts that are non-essential for survival, especially hands and feet, and implies ineffective maintenance of distal local temperature. If central temperature is preserved sufficiently but a part of the body is exposed without protection, frostbite can develop as a local injury not related to systemic defense mechanisms. In both situations, the human capacity to sense the cold and the subsequent ethological active protection responses against it are far more efficient than the physiological capacity of the organism to fight low temperatures by thermogenesis.

Cold sensing is a complex phenomenon involving diverse neural pathways, which vary depending on whether the thermal response is normal or pathological (Yin et al., 2015; Lolignier et al., 2016). In this regard, and considering normal responses to cold, we found differences in the thermal response of hands and feet after three immersions in cold water (8°C). Fingers showed greater temperatures than toes under all conditions, including basal and immersions. This agrees with previous observations, in which feet were less able to retain heat, mainly because of the greater surface area-to-mass ratio in hands than in feet, and the fact that muscular production of heat in the feet is almost absent (Johnson and Kellogg, 1985; Taylor et al., 2014). We also found a different anatomical pattern of cooling, as the thumbs were significantly more capable of maintaining heat than the other fingers. There was no parallel condition for feet, as the first toe showed lower temperatures than the fifth. This is in agreement with clinical observations of the incidence of frostbite in hands and feet, and may be useful in designing prevention strategies against frostbite regarding clothing and cold protection.

On the other hand, pathological cold pain is considered a neuropathic syndrome and involves cold hyperalgesia, pain response to innocuous temperatures and increased pain sensitivity to cold, which are a nearly universal sequelae after frostbite. In the vast majority of the cases, pathological cold pain implies nerve injury or dysfunction. The vascular system also seems to play an important role in pathological cold sensitivity. Mechanisms underlying vasoconstriction during acute cold exposures involve inhibition of the NO system in the vascular endothelium and increased sympathetic activity in the smooth muscle, with high inter-individual variability. Observations in patients with digit transplantation after trauma show that cold sensitivity is a frequent sequela, implying a persistent vasoconstriction pattern with local temperature changes (Isogai et al., 1995) regardless of the extent of nerve and vascular reconstruction. Presumably this is caused by reduced skin vessel density in the fingertips and abnormalities in vasoactive responses (Klein-Weigel et al., 2007). In the same way, botulinum toxin has been reported to be an effective treatment for frostbite sequelae, as it causes vasodilation, blocking the smooth muscle or sympathetic vasoconstriction, with clinical effects both on pain and hypersensitivity (Norheim et al., 2017).

Putting it all together, it is possible that endothelial damage secondary to frostbite may lead to abnormal vasoconstriction during cold exposure of the digits, and that the local nerve injury caused by deep freezing might play a role in the nearly universal sequelae found in these patients.

The clinical translation of the above mentioned phenomena are neurosensory (pain, cold sensitivity and numbness), vascular (increased peripheral acute vasoreactivity, changes in skin color) and musculoskeletal symptoms (joint pain) with heterogeneous manifestations and role which make it difficult to establish an universal treatment (Blair et al., 1957). In addition, cold injuries do not have to be severe to cause long-term sequelae (Taylor et al., 1989). If these alterations lead to lower digit temperatures, thus increasing the risk of re-freezing in any conditions is still unknown. In previous observations, toe stumps secondary to amputation after frostbite show lower digit temperatures when exposed to cold than healthy toes; but results regarding rewarming times are inconsistent (Morrison et al., 2015; Gorjanc et al., 2018). Conversely, our results show no significant differences between basal and maximal cooling temperatures when comparing healthy and previously frostbitten tissue. It is important to remark that amputated digits lost less temperature in our experiment than those that suffered previous frostbite but are fully conserved. This can be interpreted as a consequence of the neurosensory deficit and vascular damage derived of amputation of the non-viable tissue, which was conserved in patients that do not need surgical treatment. In these latter cases, the endothelial and neural dysfunction would remain, leading to cooler temperatures of the digits and clinical manifestations.

There are also dissimilarities to have in mind when considering the different clinical conditions in response to the topical administration of nifedipine. Nifedipine is a dihydropyridine derivative, which exhibits antagonist effects on calcium channels. Dihydropyridines are widely used as arterial-specific vasodilators of peripheral resistance arteries that block calcium influx into arterial-wall smooth muscle and cause generalized vasodilation (Weiner, 1988; Triggle, 2003; Safak and Simsek, 2006). Oral nifedipine formulations have been investigated as a treatment for diabetic ulcers, peripheral vascular diseases (Rirash et al., 2017), wound healing and hypertrophic scars (Yamamoto et al., 2010; Santis et al., 2013), with moderate evidence of effectiveness. Such oral preparations are associated with secondary systemic effects such as low systemic blood pressure and edema, and have not shown better results than placebos when treating peripheral cold-related injuries such as chilblains (Souwer et al., 2016). Therefore, we decided to use a topical Vaseline^®^-based preparation of nifedipine at 10% concentration, which we considered a sufficient dosage to induce local vascular action while avoiding clinical systemic symptoms. Digit skin temperature is correlated to skin blood flow (Rubinstein and Sessler, 1990), so measurements of skin temperature after a cold stress test are valid for evaluating the vascular response to cold. We evaluated the local temperature by means of infrared thermography, as it is widely used to assess skin temperature in various conditions and is suggested as a predictive tool to identify peripheral susceptibility to cold, in accordance with digital rewarming times (Brandstrom et al., 2008; Keramidas et al., 2014). This technique is non-invasive and avoids the use of ionizing radiation. The differences found were focused on the clinical translation of the use of nifedipine in means of temperature and vascular response, but the degree of concomitant effect of an eventual neural impairment was not elucidated and can be an interesting starting point for future observations.

Taking in to account the difference in the thermal responses of hands and feet, we analyzed the data on the response to nifedipine treatment also separately. Interestingly, we found lower basal temperatures in both healthy and amputated toes, when treated with nifedipine. Probably, the vasodilator effect of the local treatment in HD leads to skin temperature loss in a thermoneutral environment. Conversely, basal temperatures in FNA were warmer when nifedipine was applied, with significant differences in the hands. This was not reproduced in toes, which otherwise presented significantly lower temperatures in amputated limbs when using nifedipine. It is an interesting hypothesis that this effect implies that, at mild temperatures, nifedipine could be useful for those patients who previously suffered frostbite without amputation. This question could be addressed and resolved in future clinical research.

Regarding maximal cooling responses, HD were warmer when treated with nifedipine in the hands but not in the toes. FA digits showed significantly lower temperatures when treated with nifedipine. In toes, skin temperature tended toward being lower when applying nifedipine in all conditions, but only in amputated toes did this reach statistical significance.

We found topical nifedipine application can be considered as inadequate at basal temperatures, except in previously frostbitten digits which have not suffered amputation, as normal and conserved thermoregulatory systems of healthy and amputated tissues are impaired by the vasodilation effect. This can be extrapolated to those amputated toes that conserve the neurovascular system intact. At maximal cooling, whereas HD benefit from topical nifedipine, especially in the hands, amputated digits and toes universally failed to respond to the nifedipine application.

## Limitations of This Study

We have taken basal temperatures as the control condition for each subject instead of study a second control group of healthy people. Since all recruited subjects keep healthy fingers, in our opinion, the treatment with nifedipine of another group of healthy subjects would not have provided additional information. Another limitation is the small sample size (only five subjects), so further investigations are required to confirm and reinforce our findings. Finally, this study focus on the clinical impact of nifedipine regarding local temperature changes, but we cannot elucidate if the observed differences in thermal behavior are due to neurosensory or vasomotor deficits derived from previous cold damage.

## Conclusion

Topical nifedipine can be considered as a potentially interesting tool for healthy digits exposed to cold, as it leads to warmer temperatures as long as thermal insulation measures are adequate. Further research is needed to clarify whether, at mild cold temperatures, topical nifedipine application is useful in previously frostbitten tissue with no amputation. However, the potentially harmful effects of this treatment on amputations, both of hands and feet, and on healthy tissues of feet when exposed to cold, should be considered.

The differences observed on individual regional response to cold must be taken in consideration to the design of prevention strategies for subjects habitually exposed to cold environment. Thermography can be a useful, and easy to apply tool, for starting this initiative.

## Data Availability Statement

The dataset generated for this study are included in the article/Supplementary Material.

## Ethics Statement

This study was carried out in accordance with the Spanish regulations and the protocol was approved by the Institutional Ethical Committee from the University of Barcelona (Institutional Review Board number #IRB00003099). All subjects gave written informed consent and all procedures were in accordance with the Declaration of Helsinki.

## Author Contributions

AC and GV conceived the study and performed the statistical analysis of the data. AC and JG planned and carried out the experiment. JG computed the images and extracted data from the thermography images. AC took the lead in writing the manuscript. All authors contributed to the interpretation of the results, provided critical feedback on drafts, helped to shape the research report, approved the manuscript in its final form prior to submission, and contributed substantially to this report.

## Conflict of Interest

The authors declare that the research was conducted in the absence of any commercial or financial relationships that could be construed as a potential conflict of interest.
